# MetaBayesDTA: codeless Bayesian meta-analysis of test accuracy, with or without a gold standard

**DOI:** 10.1186/s12874-023-01910-y

**Published:** 2023-05-25

**Authors:** Enzo Cerullo, Alex J. Sutton, Hayley E. Jones, Olivia Wu, Terry J. Quinn, Nicola J. Cooper

**Affiliations:** 1 Biostatistics Research Group, Department of Population Health Sciences, University of Leicester, Leicester, UK; 2 Complex Reviews Support Unit, University of Leicester & University of Glasgow, Glasgow, UK; 3 Population Health Sciences, University of Bristol, Bristol Medical School, Bristol, UK; 4 Institute of Cardiovascular and Medical Sciences, University of Glasgow, Glasgow, UK

**Keywords:** Meta-Analysis, Diagnostic test accuracy, Application, Imperfect gold standard, Latent class

## Abstract

**Background:**

The statistical models developed for meta-analysis of diagnostic test accuracy studies require specialised knowledge to implement. This is especially true since recent guidelines, such as those in Version 2 of the Cochrane Handbook of Systematic Reviews of Diagnostic Test Accuracy, advocate more sophisticated methods than previously. This paper describes a web-based application - MetaBayesDTA - that makes many advanced analysis methods in this area more accessible.

**Results:**

We created the app using R, the Shiny package and Stan. It allows for a broad array of analyses based on the bivariate model including extensions for subgroup analysis, meta-regression and comparative test accuracy evaluation. It also conducts analyses not assuming a perfect reference standard, including allowing for the use of different reference tests.

**Conclusions:**

Due to its user-friendliness and broad array of features, MetaBayesDTA should appeal to researchers with varying levels of expertise. We anticipate that the application will encourage higher levels of uptake of more advanced methods, which ultimately should improve the quality of test accuracy reviews.

**Supplementary Information:**

The online version contains supplementary material available at 10.1186/s12874-023-01910-y.

## Background

### Background to meta-analysis of test accuracy

In medicine, tests are used to screen, monitor and diagnose medical conditions, and therefore it is imperative that these tests produce accurate results. This ‘accuracy’ refers to their sensitivity and specificity. The former is the probability that a test can correctly identify patients who have the disease and the latter is the probability that the test can correctly identify patients who do not have the disease. To evaluate their accuracy, studies and analyses are carried out to compare the results of the test under evaluation (called the ‘index’ test) against some existing test, which is assumed to be perfect (called the ‘reference’ or ‘gold standard’ test). Index tests typically have lower accuracy than the gold standard; however, they are often quicker, cheaper and/or less invasive.

Standard methods for the meta-analysis of test accuracy assume that the gold standard test is perfect - i.e., that the test is 100% sensitive and specific. These models dichotomize the data into diseased and non-diseased according to the results of the reference test, and include the bivariate model of Reitsma et al. [1] and the hierarchical summary receiver operating characteristic (HSROC) model of Rutter & Gatsonis [2]. These models have been shown to be equivalent in practice when no covariates are included [3]. Models which do not assume a perfect gold standard have also been developed [4–6]. These models - which are often referred to as *latent class models* (LCMs) - assume that each test is measuring the same latent disease, and each individual is assumed to belong in either the diseased or non-diseased classes. These methods can also model the correlation between each test within each disease class (i.e. the *conditional dependence* between tests). All of the aforementioned methods take into account the correlation between sensitivity and specificity across studies.

### Why is this application needed?

The models discussed in the previous section require statistical programming expertise using software such as R or Stata. Cochrane, an organisation who help support evidence-based decisions about health interventions such as diagnostic and screening tests. Whilst they do provide free software RevMan [7] using the Moses-Littenberg method [8], it fails to appropriately account for random effects and the correlation between sensitivity and specificity across studies. Carrying out meta-analysis of test accuracy using online applications has a lower user burden since no programming is needed. Not only does this make such methods accessible to a broader array of people, it also streamlines the workflow for more experienced data analysts.

Other web applications for the meta-analysis of test accuracy include MetaDTA [9–11] and BayesDTA [12]. The former uses frequentist methods and implements the bivariate model [1], allowing for risk of bias and quality assessment data to be incorporated into the results plots. The latter uses Bayesian methods and incorporates both the bivariate model [1] and the LCM model [4, 5]. Similarly to BayesDTA, our application, MetaBayesDTA [13], runs Bayesian versions of both the bivariate [1] and the LCM model [4, 5], and is powered by Stan [14], a Bayesian model fitting software. However, unlike BayesDTA, our application can also conduct subgroup analysis and meta-regression for the bivariate model, and can be used to conduct a comparative meta-analysis of test accuracy for 2 or more tests using categorical meta-regression (assuming the same variances between tests), using methods recommended in chapter 11 of version 2 of the Cochrane handbook for systematic reviews of diagnostic test accuracy [15]. Furthermore, for the LCM model, rather than assuming all studies use the same reference tests, it can model multiple reference tests. It also allows users to compare the fit between different LCM models. A full comparison between MetaBayesDTA, MetaDTA and BayesDTA is shown in Table 1.

**Table 1:** Table comparing features of MetaBayesDTA, MetaDTA and BayesDTA

	MetaBayesDTA	MetaDTA	BayesDTA
Bayesian or frequentist	Bayesian	Frequentist	Bayesian
Model assuming gold standard (bivariate model)	$\checkmark$	$\checkmark$	$\checkmark$
For bivariate - subgroup analysis	$\checkmark$	$\times$	$\times$
For bivariate - univariate meta-regression	$\checkmark$	$\times$	$\times$
For bivariate - comparative accuracy of 2+ tests	$\checkmark$	$\times$	$\times$
Model not assuming perfect gold standard (LCM)	$\checkmark$	$\times$	$\checkmark$
For LCM - model multiple reference tests	$\checkmark$	$\times$	$\times$
For LCM - subgroup analysis	$\times$	$\times$	$\times$
For LCM - univariate meta-regression	$\times$	$\times$	$\times$
For LCM - comprehensive assessment of model fit	$\checkmark$	$\times$	$\times$
Interactive layout / pop-up menus	$\checkmark$	$\times$	$\times$
Risk of bias and quality assessment on plots	$\checkmark$	$\checkmark$	$\times$
Appropriate restrictions in place	$\checkmark$	$\times$	$\times$

## Implementation

### Aims

Our objective was to make a web application which would be accessible to a wide variety of researchers and enable them to conduct a robust Bayesian statistical analysis for meta-analysis of test accuracy - including subgroup analysis, meta-regression, comparative test accuracy, and the ability to conduct meta-analysis of test accuracy without assuming a perfect reference test. This would all be possible despite the researcher not possessing sufficient experience in R [16] and Stan [14]. It is also aimed at researchers who can use R and/or Stan (e.g. some data analysts, statisticians, clinical researchers, etc) but would still want to use a web application for efficiency.

### Software

We used the statistical programming language R [16] to create our web application, using a variety of packages. One such package includes Shiny [17], which enables R users to create web applications without having to have knowledge of web development languages such as HTML and JavaScript. Another package used includes rstan [18], which enables users to fit Bayesian statistical models in R using Stan [14], and is what we used to fit both the bivariate and LCM models in the application. A new user interface format was developed using the R packages shinydashboard [19] and shinywidgets [20]. This allows the app to have a clean layout, with many of the menus hidden unless the user chooses to display them.

## Results

In this section, we will demonstrate the application through a motivating example dataset containing a total of 13 studies from a Cochrane meta-analysis [21], which assessed the accuracy of the Informant Questionnaire on Cognitive Decline in the Elderly (IQCODE) - a screening test used to detect adults who may have clinical dementia within secondary care settings.

### Data

**Fig. 1:**
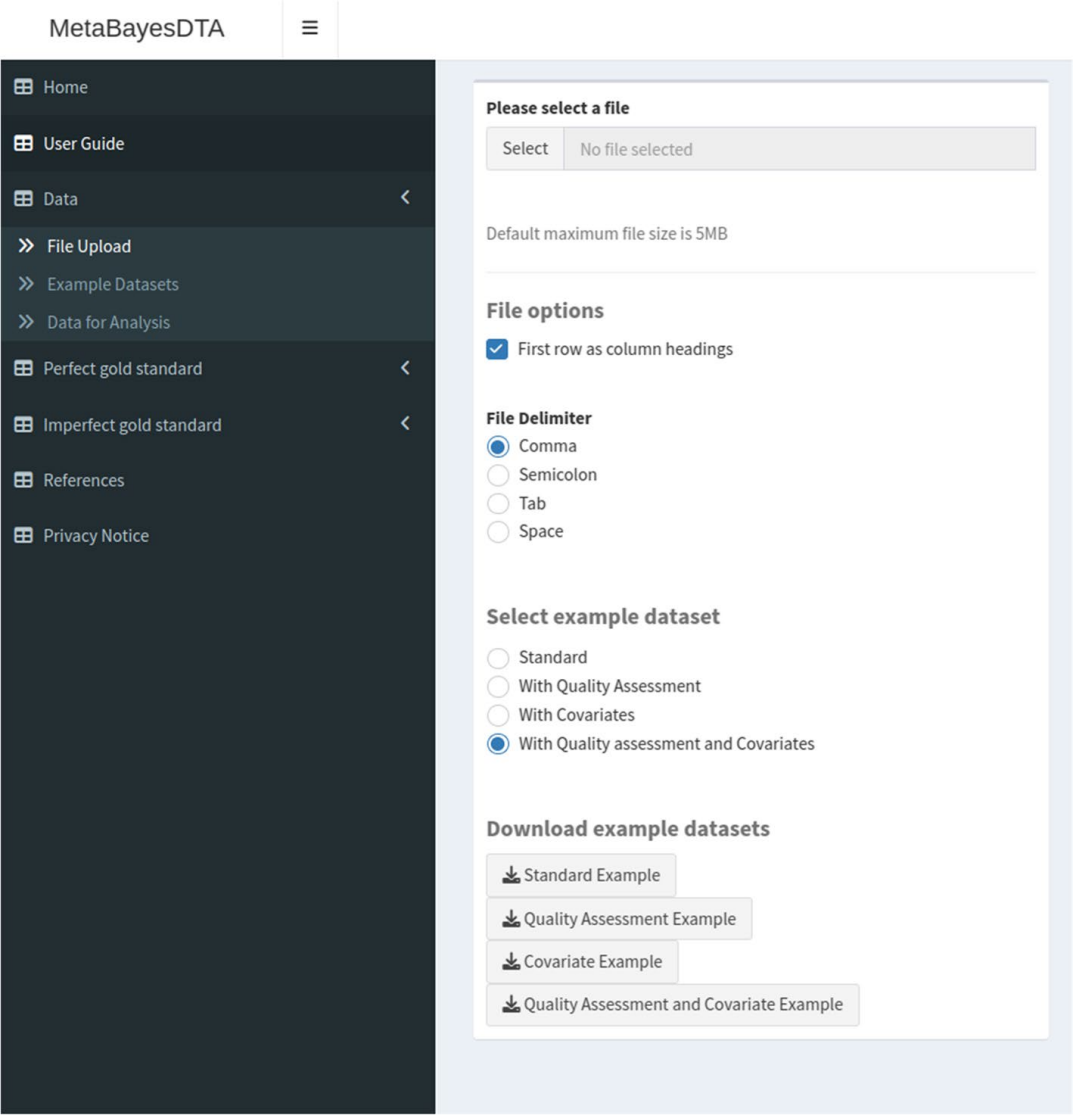
Screenshot of ‘Data’ tab, showing part of the ‘File Upload’ subtab

The ‘Data’ tab (see Fig. 1) allows users to upload their data. The number of columns the datasets must have will vary depending on whether quality assessment data and/or covariate data is included. Datasets involving no quality assessment or covariate data will have six columns, those involving quality assessment data thirteen, those involving covariates at least seven, and those involving both quality assessment and covariate data at least fourteen. The quality assessment data which can be included is from quality assessment carried out using the QUADAS-2 (QUality Assessment of Diagnostic Accuracy Studies, version 2) tool [22]. This tool has four domains: (i) patient selection, (ii) index test, (iii) reference standard and (iv) flow of patients through the study and timing of the index test(s) and reference standard.

The ‘File Upload’ subtab is pre-loaded with an example dementia dataset from the Cochrane meta-analysis [21], which is described in more detail in the ‘Example datasets’ subtab in the application. The ‘Data for Analysis’ subtab shows the dataset currently being used.

We will use this dataset to demonstrate the application throughout the remainder of this section. To analyse the data, the Cochrane meta-analysis [21] used the bivariate model and found a pooled summary estimates of 0.91 (95% CI [confidence interval] = (0.86, 0.94)) and 0.66 (95% CI = (0.56, 0.75)) for the sensitivity and specificity, respectively.

### Perfect gold standard

The ‘Perfect gold standard’ page consists of three tabs: meta-analysis, meta-regression and subgroup analysis. All three tabs use the bivariate model proposed by Reitsma et al. [1], employing the variation which uses binomial likelihoods proposed by Chu and Cole [23].

#### Meta-analysis

The Meta-analysis subtab is split into two halves - the left half consists of the following tabs: ‘priors’, ‘run model’, ‘study-level outcomes’, ‘parameter estimates’, ‘parameters for RevMan’, and ‘model diagnostics’. The right half has the tabs ‘sROC [summary Receiver Operating Characteristic] plot’, ‘Forest Plots’ and Prevalence’.

**Fig. 2:**
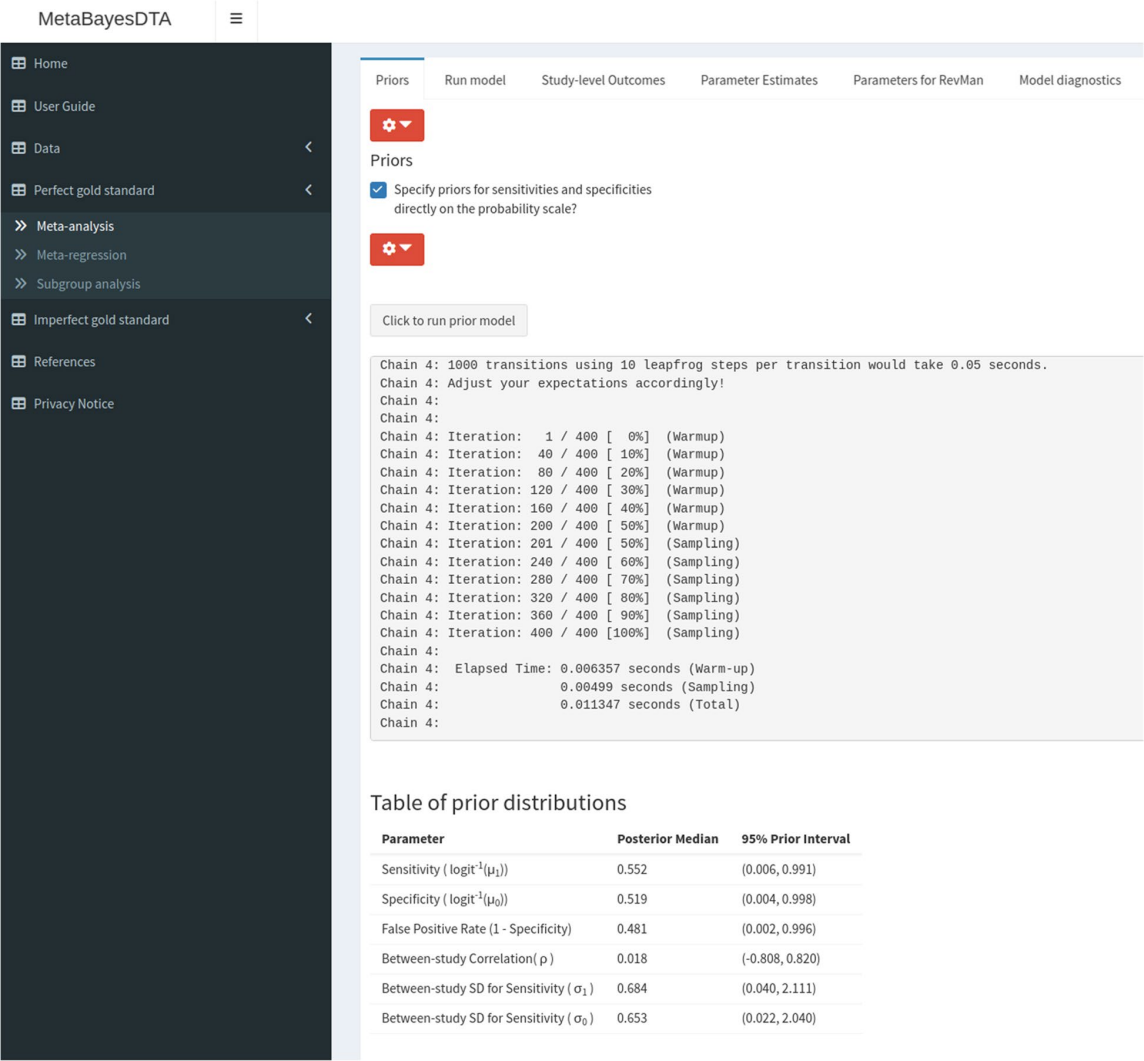
Screenshot of ‘Perfect gold standard’ tab, ‘Priors’ subtab within ‘Meta-analysis’ subtab

Since all of the models in the app are Bayesian, prior distributions need to be specified. The ‘priors’ subtab (see Fig. 2) is where users specify prior distributions. The priors can be changed if some information is known about them, and they can be specified in terms of the logistic-transformed (“logit”) sensitivity and specificity, or directly on the probability scale. The default prior distributions are weakly informative. More specifically, for the pooled logit sensitivity and logit specificity, we used a normal distribution with mean zero and SD of 1.5 (*N*(0, 1.5)), which is equivalent to a 95% prior interval (that is, the interval formed by the 2.5% and 97.5% quantiles of the prior distribution) of (0.05, 0.95) on the probability scale. For the between-study SD’s (standard deviations) we used a truncated (at zero) normal with zero mean and unit SD ($N_{ \ge 0 }(0, 1)$). This prior allows for a very large amount of between-study heterogeneity if the data demands; for example, if the pooled sensitivity is found to be 0.80, then this prior assumes that the study-specific sensitivities will be in the range (0.069, 0.996) with 95% probability. Finally, for the between-study correlation we used an LKJ (Lewandowski-Kurowicka-Joe) [24] prior with shape parameter of 2 (*LKJ*(2)), which gives a 95% prior interval of $(-0.8, 0.8)$. In general, we suggest leaving all of these prior distributions to the defaults. However, if it is known that the sensitivity or specificity for the test under evaluation may be very high (e.g. $> 95\%$), then a prior which places more prior probability on these values would be more appropriate than the default *N*(0, 1.5) prior - for instance a prior of *N*(3, 1.5) which would be equivalent to a 95% prior interval of (0.500, 0.998) on the probability scale.

Users can examine the prior distributions specified by clicking on the button ‘Click to run prior model’ and the prior medians and 95% prior intervals are shown in a Table (see bottom of Fig. 2). Plots of the prior distributions are also displayed (below the table - not shown in Fig. 2).

**Fig. 3:**
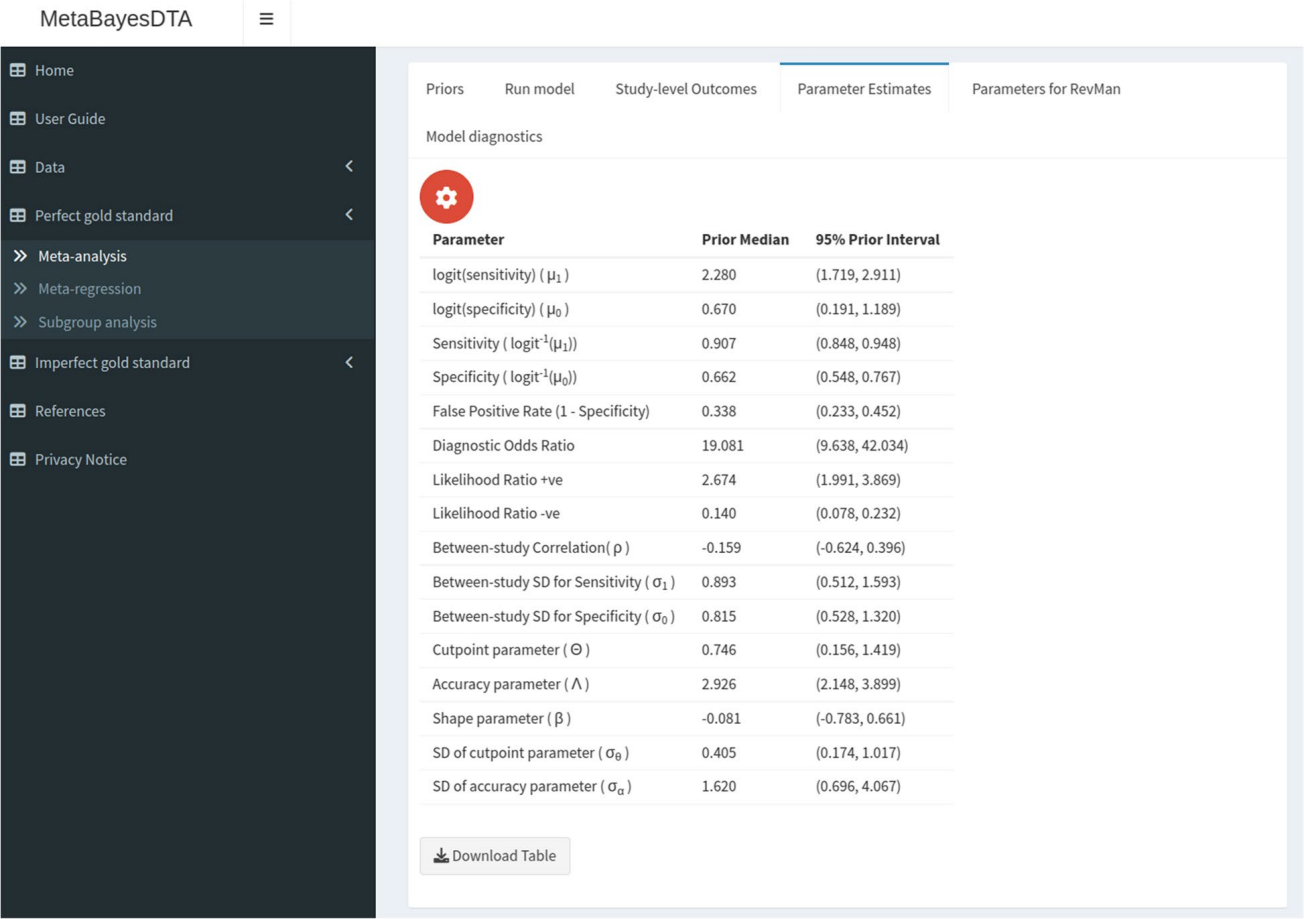
Screenshot of ‘Perfect gold standard’ tab, ‘Parameter estimates’ subtab within ‘Meta-analysis’ subtab

Users can run the model by clicking on the ‘Click to run model’ button within the ‘Run model’ subtab. In this subtab, users can also run sensitivity analysis - more specifically, this is where any number of studies can be excluded from the analysis to assess the influence of particular studies on the overall pooled estimates.

The ‘study-level outcomes’ subtab displays key study information that is also displayed in the ‘Data’ tab, as well as the sensitivity and specificity in each study and study weights - that is, the amount that each study contributes to the overall sensitivity and specificity estimate, calculated using the method from Burke et al. [25]. The ‘parameter estimates’ subtab (see Fig. 3) consists of a table with the posterior medians and 95% posterior intervals (otherwise known as credible intervals [CrI’s]) for key summary parameters including logit sensitivities and specificities, diagnostic odds ratio and likelihood ratios, between-study correlation and standard deviations, and HSROC parameters. The HSROC parameters are estimated from the bivariate model parameters using the relations shown in Harbord et al. [3].

The ‘parameters for RevMan’ subtab consists of the parameter estimates (posterior medians) needed by Cochrane’s RevMan software to build ROC plots for people who want to include the analysis results in a Cochrane review. The ‘Model diagnostics’ subtab contains important diagnostics that users must check to ensure whether the model is valid. These include the Stan sampler diagnostics [14, 26] - divergent transitions and iterations which have exceeded the maximum treedepth (these should both be 0), split R-hat statistics (should be less than 1.05), and posterior density and trace plots [14].

**Fig. 4:**
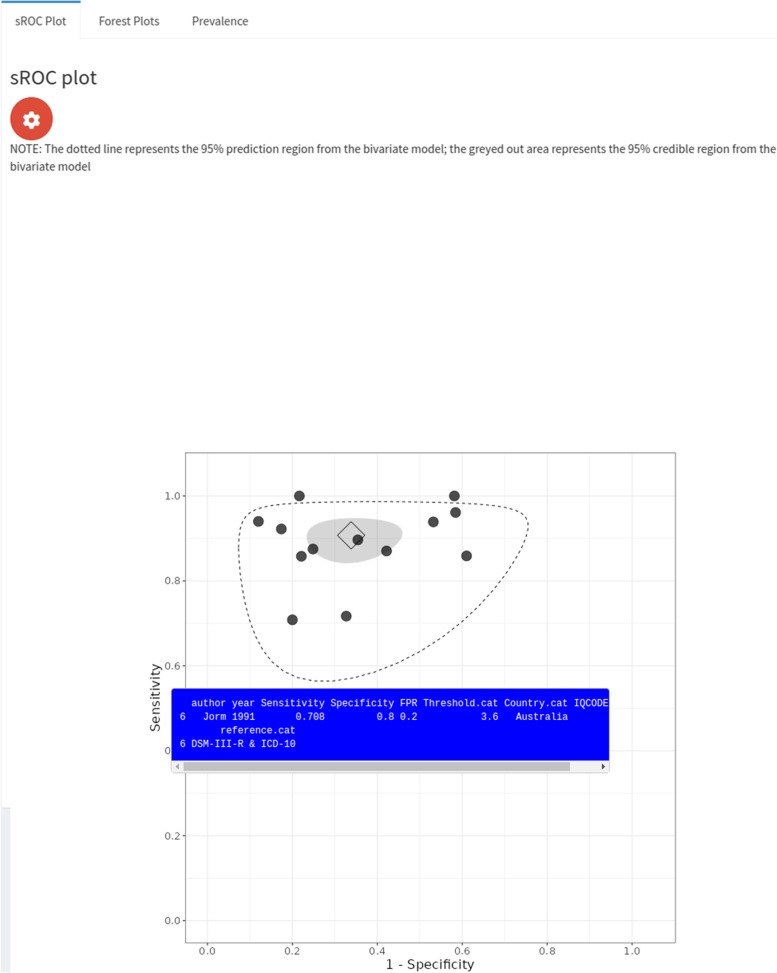
Screenshot of ‘Perfect gold standard’ tab, ‘sROC Plot’ subtab within ‘Meta-analysis’ subtab. The blue box contains information for the study (Jorm et al) corresponding to the point on the bottom-left, and appears when the user clicks on this point

The sROC plot is displayed in the ‘sROC plot’ subtab (see Fig. 4). This plot displays the summary estimates, 95% credible and prediction regions and study-specific sensitivities and specificities. The plot has a range of customization options; for instance, it allows users to change the size of the summary estimates and study-specific points, display the sROC curve, disease prevalence and percentage study weights of each study. It is also interactive - users can click on the study-level points and study-level information will appear over the plot - this is demonstrated in Fig. 4, where the bottom-left point corresponding to the Jorm et al. [27] study has been clicked on. This plot, as well as the other plots produced by the application, can be downloaded. Risk of bias and quality assessment information, if available in the dataset, can also be displayed on the plot (see supplementary material Fig. 1).

The ‘forest plots’ subtab contains the forest plots - which are plots showing the sensitivity and specificity in each study as well as the corresponding 95% confidence intervals. The ‘prevalence’ subtab contains a tree diagram which puts the summary estimates into context - it shows how many patients would test positive and negative for a given disease prevalence, and then out of those who test positive and negative, which are diseased and non-diseased. There is also another tree diagram option, which first splits the population by disease status and then by test result.

We analysed the IQCODE dementia dataset discussed previously using our application, using the Bayesian bivariate model assuming a perfect gold standard. We used the default prior distributions (see Fig. 2), and obtained virtually the same results as the frequentist analysis conducted in the original study - sensitivity and specificity estimates of 0.91 (95% credible interval [CrI] = (0.85, 0.95)) and 0.66 (95% CrI = (0.55, 0.77)), respectively (see Fig. 3). An sROC plot showing the results is shown in Fig. 4.

#### Meta-regression

The ‘Meta-regression’ tab is where users can run the bivariate model including a categorical or continuous covariate in an attempt to explain any between study heterogeneity, and consists of subtabs similar to the ‘Meta-analysis’ tab. The ‘Run model’, ‘study-level outcomes’, ‘Model Diagnostics’ and ‘sROC plot’ subtabs are the same as those in the ‘Meta-analysis’ tab.

Rather than a ‘priors’ subtab, it has a ‘Model set up & priors’ tab, since users also need to select the covariate to use. Furthermore, if using a continuous covariate, users need to specify the value to use for centering (the default is the mean of the values of the covariate) and which value of the covariate to calculate the summary accuracy estimates at. For the default priors, for continuous meta-regression we used *N*(0, 1.5) priors for the pooled logit sensitivity and specificity intercepts and *N*(0, 1) priors for the pooled logit sensitivity and specificity coefficients. For categorical meta-regression, we used *N*(0, 1.5) priors for the pooled logit sensitivities and specificities for each level of the covariate. For both continuous and categorical meta-regression, similarly to the model with no covariates, we used ($N_{ \ge 0 }(0, 1)$) and *LKJ*(2) priors for the between-study SD’s and correlations, respectively. In general, we suggest leaving these priors at the default values in most cases. However, sometimes it will make sense to change them. For example, as we mentioned in the “Meta-analysis” section previously, for categorical meta-regression, if it is known that the sensitivity or specificity for the test under evaluation may be very high $( > 95\% )$, then a prior which places more prior probability on these values would be more appropriate than the default *N*(0, 1.5) prior. For continuous meta-regression, the default *N*(0, 1) prior for the coefficient terms will generally allow the coefficient to have a large influence if the data allows. However, for coefficients which are on a small scale, such as disease prevalence, it might make more sense to try priors which are less informative than the default. For example, if the covariate is (centered) disease prevalence and the mean value of the disease prevalence is 0.10, and the sensitivity at this value is found to be 0.80, then this prior will assume that the value of sensitivity for a 10% increase in disease prevalence (i.e. a prevalence of 0.20) is in the interval (0.77, 0.83) with 95% probability, whereas a prior of *N*(0, 5) will assume an interval of (0.60, 0.92) with 95% probability. In this case, the latter would be more appropriate than the default prior if disease prevalence is thought to be (or if it cannot be ruled out to be) strongly (or negatively) associated with test accuracy.

**Fig. 5:**
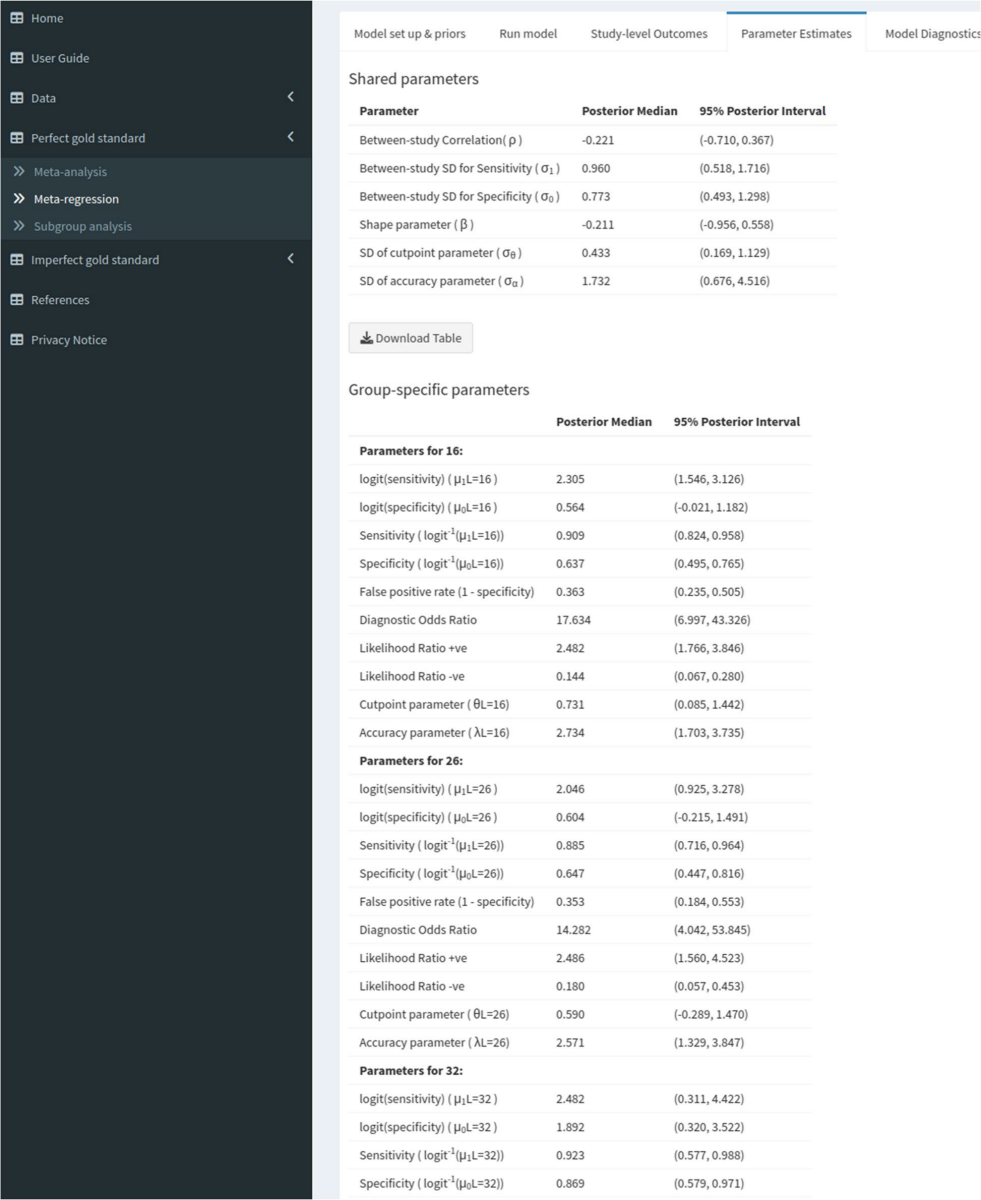
Screenshot of ‘Perfect gold standard’ tab, ‘parameter estimates’ subtab within the ‘meta-regression’ subtab

**Fig. 6:**
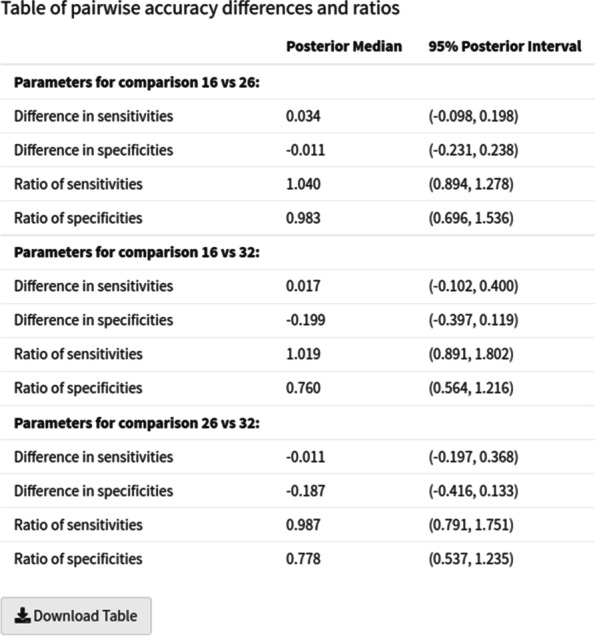
Screenshot of the table of pairwise accuracy differences and ratios table; in the ‘parameter estimates’ subtab within the ‘meta-regression’ subtab

The ‘parameter estimates’ subtab contents will vary depending on whether continuous or categorical meta-regression is being carried out. For the continuous meta-regression, there will be one table showing the parameters which do not vary, regardless of what the user chooses for the covariate value to calculate the summary estimates at, and another table containing the parameters that do vary. For categorical meta-regression (see Fig. 5), there will be one table containing the parameters shared between studies, such as between-study correlation and standard deviations, and another table showing the group-specific parameters, such as the sensitivity and specificity at each level (i.e. group) of the categorical covariate. Furthermore, there will also be a table which displays the pairwise differences and ratios between the pooled sensitivity and specificity estimates (see Fig. 6).

The ‘accuracy vs covariate’ subtab contains a plot which displays the summary sensitivity and specificity posterior medians and 95% credible intervals against the selected covariate. For categorical meta-regression, there will be a posterior median and 95% credible interval for each category of the covariate, whereas for continuous meta-regression there is a smooth line corresponding to the 95% posterior median and 95% credible interval bands as the covariate spans its observed range.

**Fig. 7:**
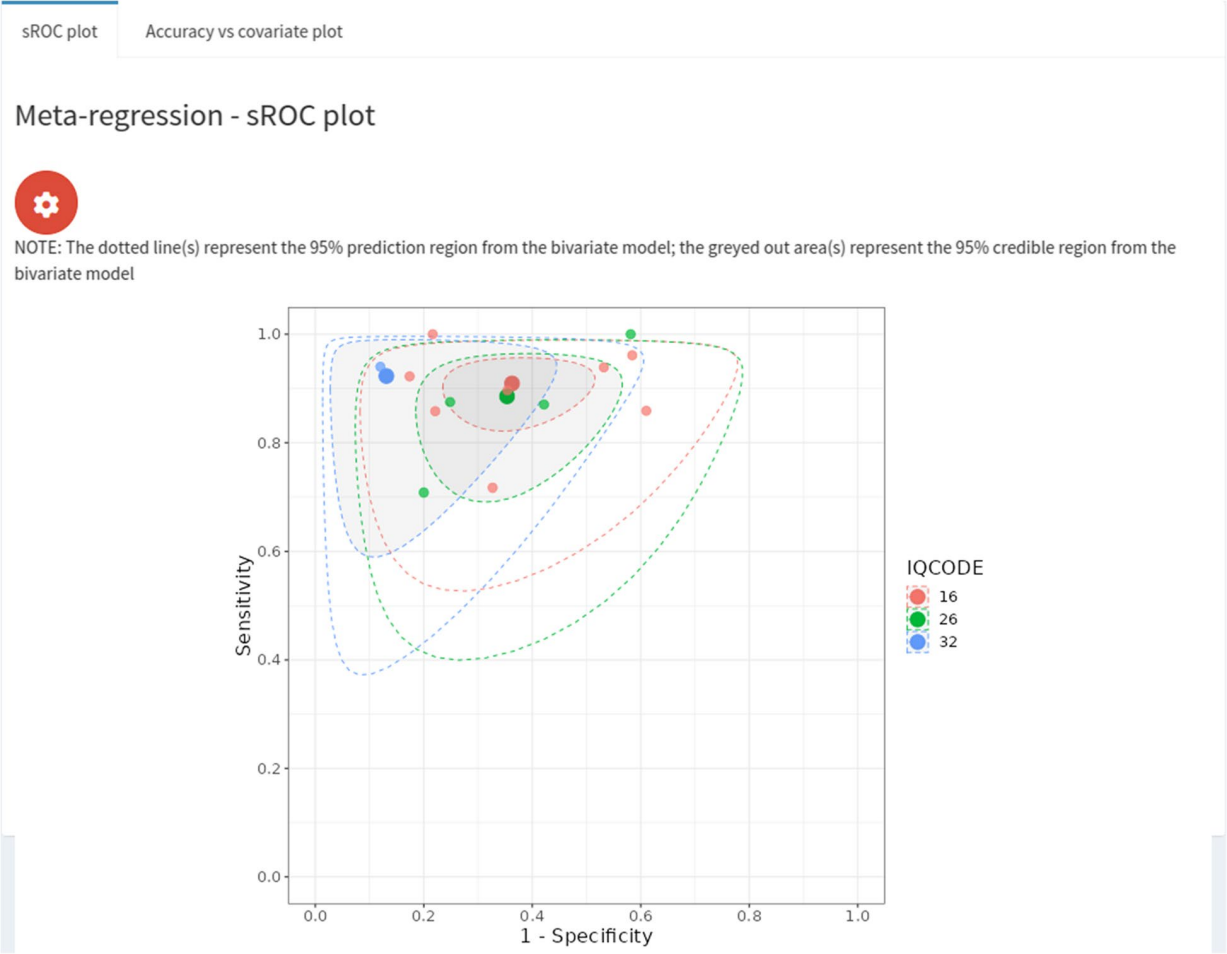
Screenshot of ‘Perfect gold standard’ tab, ‘sROC plot’ subtab within ‘Meta-regression’ subtab

We conducted a categorical meta-regression using the type of IQCODE test (either the 16, 26 or 32-item version) used as the covariate. The results for the 16-item and 26-item groups were very similar (see Fig. 5) - for the 16-item group we obtained sensitivity and specificity estimates of 0.91 (95% CrI = (0.82, 0.96)) and 0.64 (95% CrI = (0.50, 0.77)). For the 26-item group we obtained sensitivity and specificity estimates of 0.89 (95% CrI = (0.72, 0.96)) and 0.65 (95% CrI = (0.45, 0.82)). For the 32-item group, we obtained a similar sensitivity - 0.92 (95% CrI = (0.58, 0.99), but for the specificity we obtained a very different result - 0.87 (95% CrI = (0.58, 0.97)) - however, this was only based on 1 study. Looking at the pairwise differences (see Fig. 6), we can see that the 95% credible intervals contain 0 for all of the sensitivities and specificities - indicating that none of the differences are significant - even for the comparison to the 32-item group, despite the posterior medians being relatively large. Similarly, the pairwise ratio’s all contain 1, implying that none of them are significant. An sROC plot showing the results is shown in Fig. 7.

#### Subgroup analysis

Our app also allows users to run subgroup analyses for categorical covariate data. This will run a separate bivariate meta-analysis for each subgroup, obviating the need for users to partition their data and run the analysis multiple times. Such analyses differ from including the subgrouping variable as a categorical covariate and using the regression facility outlined above in the previous section, because here separate random effect variances are calculated for each group, whereas they are assumed to be the same and estimated jointly in the regression. The ‘subgroup analysis’ tab contains the same subtabs as the ‘meta-regression’ tab, and the subtabs will look mostly the same as when running a categorical meta-regression. The key difference is that in the ‘parameter estimates’ subtab, there is just one table showing the parameters for each subgroup, since there are no parameters shared between the subgroups. For the prior distributions, similarly to the standard meta-analysis model, we recommend keeping them at the default values in most cases. However, sometimes it will make sense to change them. For example, it is known that the sensitivity or specificity for the test under evaluation may be very high $( > 95\% )$, then a prior which places more prior probability on these values would be more appropriate than the default *N*(0, 1.5) prior.

**Fig. 8:**
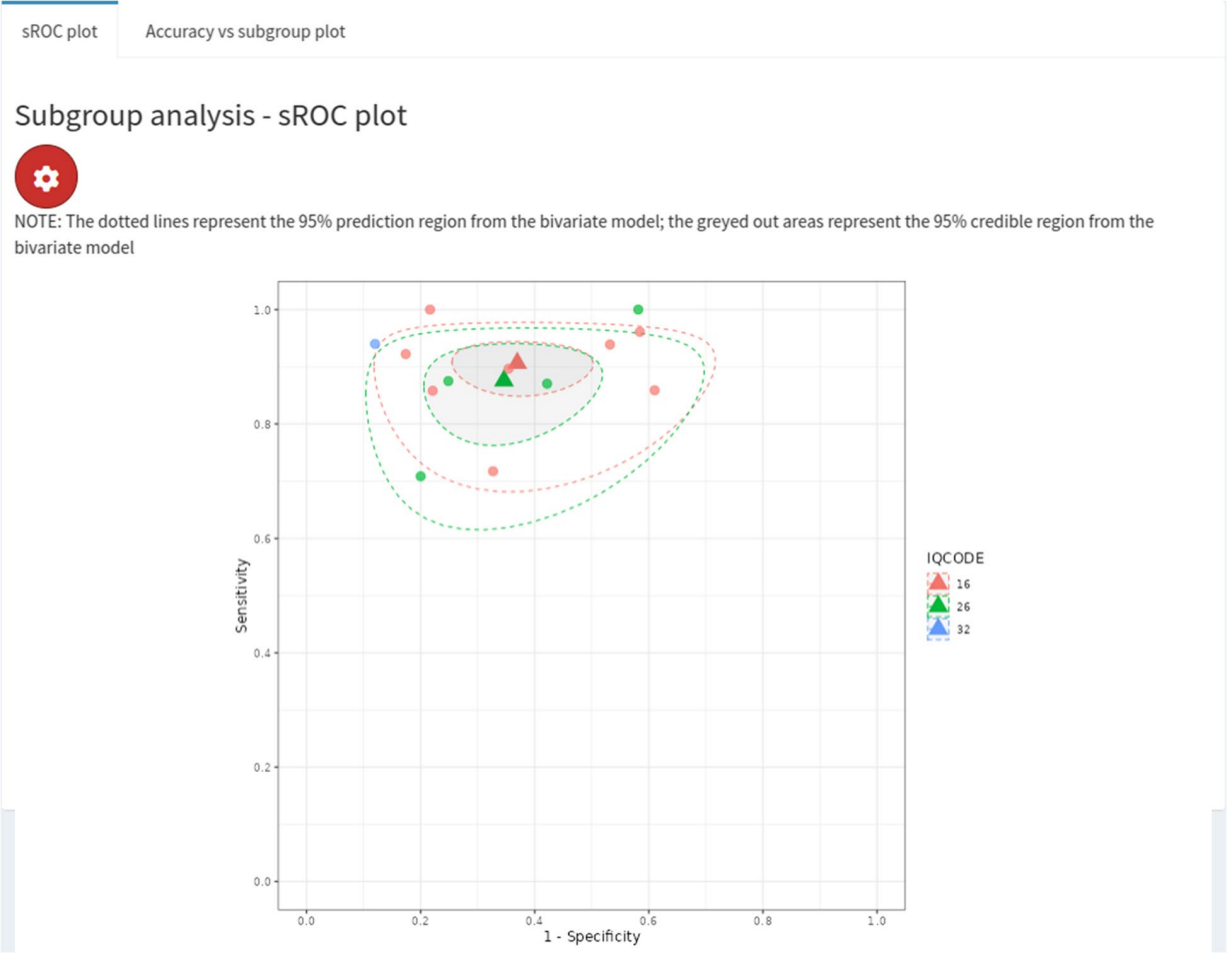
Screenshot of ‘Perfect gold standard’ tab, ‘sROC Plot’ subtab within ‘subgroup analysis’ subtab. Plot corresponds to subgroup analysis for IQCODE test type

We conducted a subgroup analysis for the type of IQCODE test used - either the 16, 26 or 32-item version. Only one study used the 32-item version, so no analysis could be conducted for this subgroup. Four studies used the 26-item version and eight studies used the 16-item version. The results for these two subgroups were very similar. More specifically, for the 26-item subgroup, we obtained sensitivity and specificity estimates of 0.88 (95% CrI = (0.77, 0.94)) and 0.65 (95% CrI = (0.49, 0.79)), and for the 16-item subgroup we obtained sensitivity and specificity estimates of 0.91 (95% CrI = (0.86, 0.95)) and 0.63 (95% CrI = (0.51, 0.74)). These results can be compared to the regression demonstration in the previous section, which made the stronger assumption that the between-study heterogeneity levels are the same across groups. An sROC plot showing the results of the subgroup analysis is shown in Fig. 8.

### Imperfect gold standard

In addition to meta-analysis of test accuracy which assumes a perfect gold standard using the bivariate model discussed in the “perfect gold standard” section, our app also allows users to run meta-analysis of test accuracy without assuming a perfect gold standard using LCMs [4, 5] within the “Imperfect gold standard” tab. This tab has the following subtabs: ‘model set up & priors’, ‘Run model’, ‘study-level outcomes’, ‘parameter estimates’, ‘model diagnostics’, and ‘sROC plot’.

**Fig. 9:**
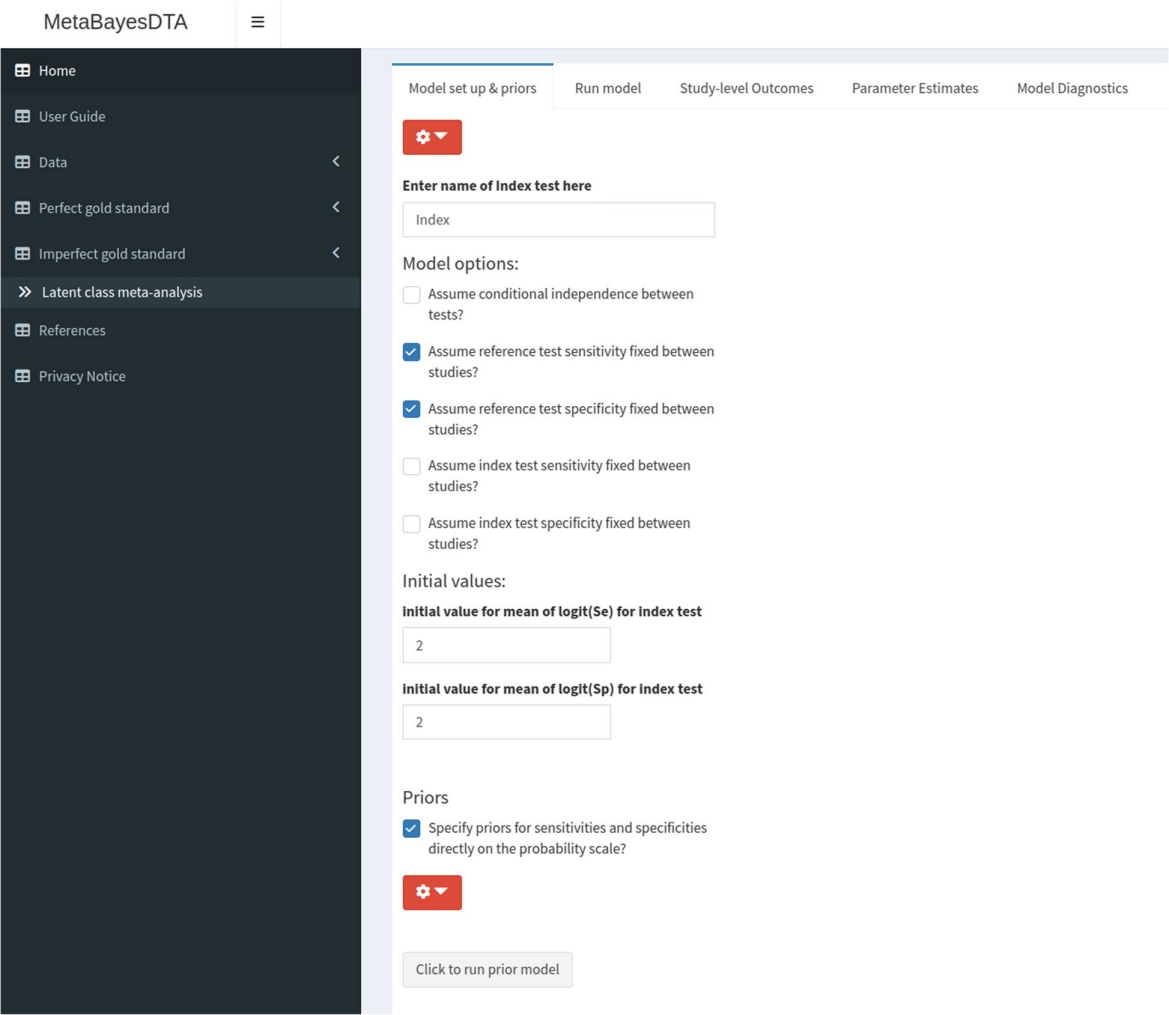
Screenshot of ‘Imperfect gold standard’ tab, ‘Model set up & priors’ subtab within ‘Latent class meta-analysis’ subtab

The ‘Model set up & priors’ subtab for the LCM has more options than that of the bivariate model (see Fig. 9). This is because, in contrast to the bivariate model, which only estimates accuracy for the index test, the LCM model estimates accuracy for both the index and the reference test(s), as well as the disease prevalence in each study. Users can choose various modelling options - more specifically, they can choose whether the reference and index test sensitivities and specificities are fixed between studies (i.e. “fixed effects”), or whether they can vary between studies (i.e. “random effects”). They can also choose whether to assume * conditional independence* between tests. In practice, the conditional independence assumption is typically not a reasonable assumption to make, since it assumes that the test results are uncorrelated within the diseased and non-diseased groups [28]. However, sometimes it is not possible to run a model which does not assume conditional independence because it might be *nonidentifiable* [29]; that is, there might be two (or more) sets of parameter values that fit the data equally well. For instance, the model may estimate the sensitivity of a test to be equal to *both* 0.20 and 0.80. This is more likely to occur when the the number of parameters being estimated from our model is greater than what is possible for the given dataset (although it can also occur when it is possible to estimate all parameters). One way to lower the chance of this happening is to introduce more informative prior information - for instance, information about the accuracy of the reference test(s) is often known and can be obtained by searching the relevant literature and by consulting clinicians. Therefore, we would recommend using prior distributions based on such data as opposed to the default *N*(0, 1.5) priors for the logit-transformed specificities and sensitivities. We would generally suggest to leave the other priors at the default values.

In addition to the Stan sampler diagnostics, R-hat statistics, posterior density and trace plots, the ‘Model diagnostics’ subtab has two plots which allows users to assess the fit of the model - the correlation residual plot [30] and the frequency table probability residual plot. It also has a table which shows the overall deviance and study-specific deviances.

We conducted an analysis using LCM models which do not assume a perfect gold standard. The studies included a variety of reference standards - four studies used the Diagnostic and Statistical Manual of Mental Disorders version III, revised (DSM-III-R) [31]; seven studies used version IV (DSM-IV) [32]; one study used the National Institute of Neurological and Communicative Diseases and Stroke/Alzheimer’s Disease and Related Disorders Association (NINCDS-ADRDA) [33] criteria; and one study used a combination of the DSM-III-R [31] and the International Classification of Diseases, version 10 (ICD-10) [34] criteria. Rather than assuming all the reference tests have the same accuracy (as is commonly done in practice), our application allows us to model the differences between the various reference tests using meta-regression. To incorporate prior knowledge into the model, we used information from an umbrella review [35] (i.e., a review of systematic reviews and meta-analyses). This umbrella review found that the accuracy for clinical dementia diagnostic criteria had a sensitivity range of 0.53-0.93 and a specificity range of 0.55-0.99. For the sensitivities and specificities of all of the reference tests, we used priors corresponding to a 95% prior interval of (0.43, 0.96).

#### Analysis assuming conditional independence

**Fig. 10:**
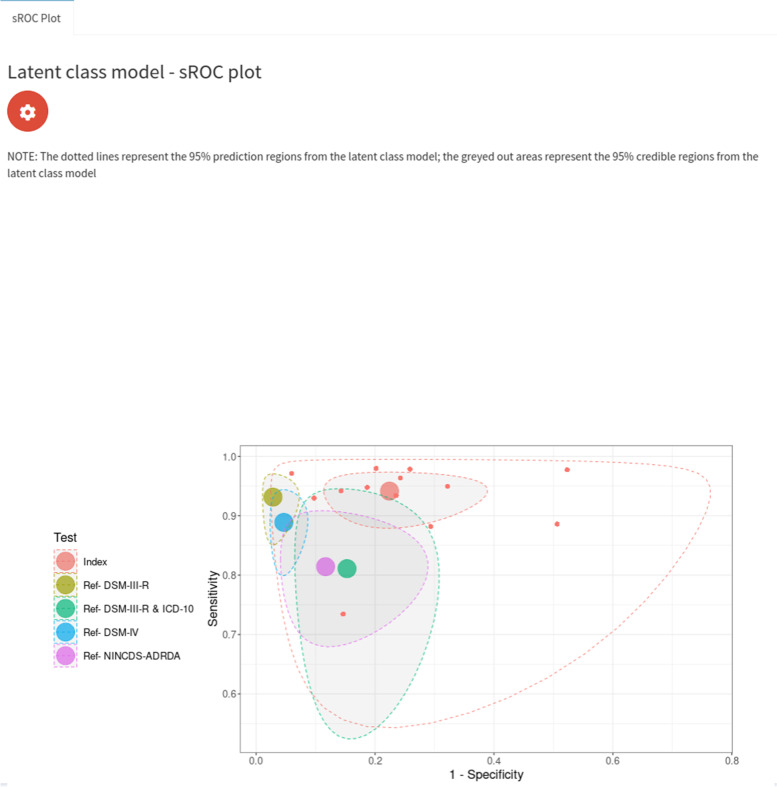
sROC plot for LCM analysis, assuming conditional independence between the IQCODE and reference tests, fixed effects for the reference tests, and random effects for the IQCODE. The red dots correspond to the study-specific estimates for the IQCODE

**Fig. 11:**
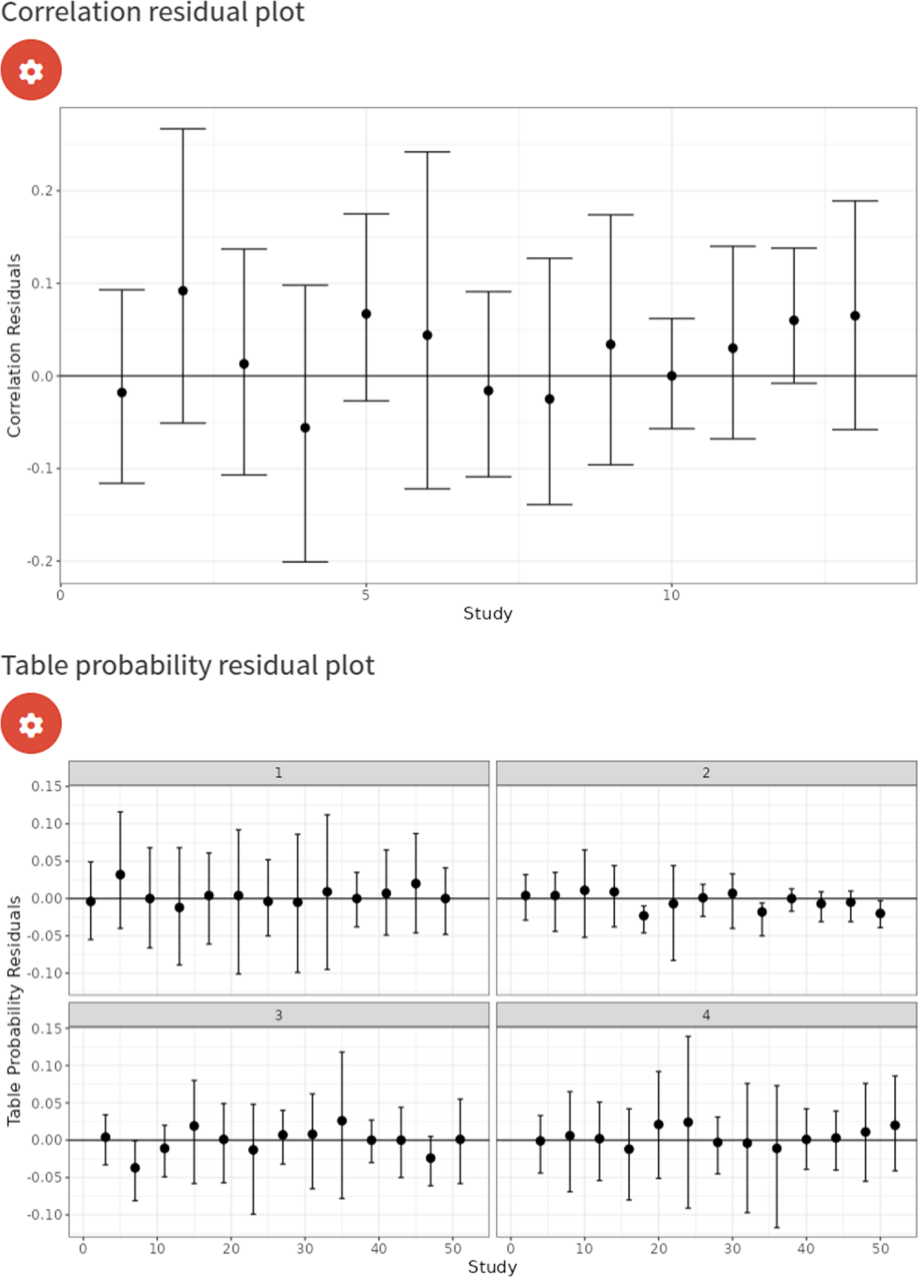
Correlation residual plot for LCM analysis, assuming conditional independence between the IQCODE and reference tests, fixed effects for the reference tests, and random effects for the IQCODE

We first analysed the data using a model which assumed conditional independence between the index test (IQCODE) and the reference tests. We assumed that the reference tests were fixed between studies and assumed random effects for the IQCODE. For the IQCODE, we obtained sensitivity and specificity estimates of 0.94 (95% CrI = (0.89, 0.98)) and 0.77 (95% CrI = (0.62, 0.89)). The IQCODE was estimated to have a higher sensitivity but lower specificity than all of the reference tests. These results suggest that the analysis assuming a perfect gold standard conducted previously underestimates the sensitivity of the IQCODE by around 3% and underestimates the specificity by around 11%. An sROC plot of the results is shown in Fig. 10. The posterior distribution plots (see supplementary material Fig. 2) are satisfactory for all parameters since they are all unimodal (i.e. they all have one peak) and the trace plots are also satisfactory for all parameters since they indicate that the chains overlap considerably and hence have mixed well (see supplementary material Fig. 3). Furthermore, all other sampler diagnostics were satisfactory (i.e., all R-hat statistics were less than 1.05 and there were no divergent transitions or any iterations which exceeded the maximum treedepth [14]). Attempts to run a model assuming conditional independence between tests with random effects for the reference tests and the index test resulted in unsatisfactory posterior distributions plots (see supplementary material Fig. 4). More specifically, some of the posterior distribution plots for the accuracy parameters were bimodal - that is, they have two peaks, which means they would estimate the accuracy as being two different values, indicating that the model is non-identifiable. The correlation residual plot (see top of Fig. 11) suggests the conditional independence model provides a satisfactory fit to the data, since all of the 95% CrI’s cross the zero line. However, whilst overall good, the frequency table probability residual plot (see bottom plot of Fig. 11) shows that the 95% CrI’s of 4 studies do not overlap the zero line. We found the median and mean overall deviance of this model to be 54.8 and 54.5, respectively.

#### Analysis assuming conditional dependence

**Fig. 12:**
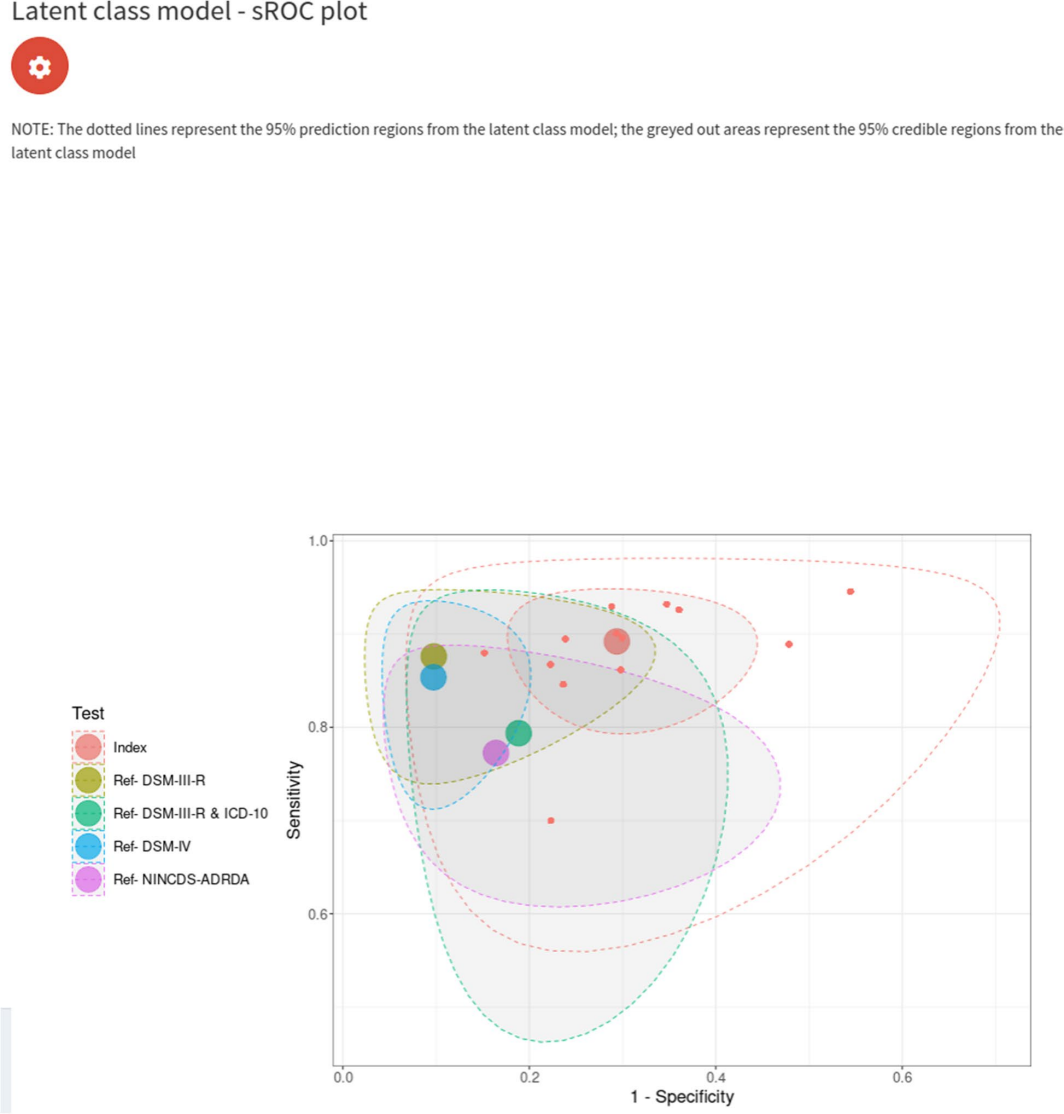
sROC plot for LCM analysis, assuming conditional dependence between the IQCODE and reference tests, fixed effects for the reference tests, and random effects for the IQCODE. The red dots correspond to the study-specific estimates for the IQCODE

**Fig. 13:**
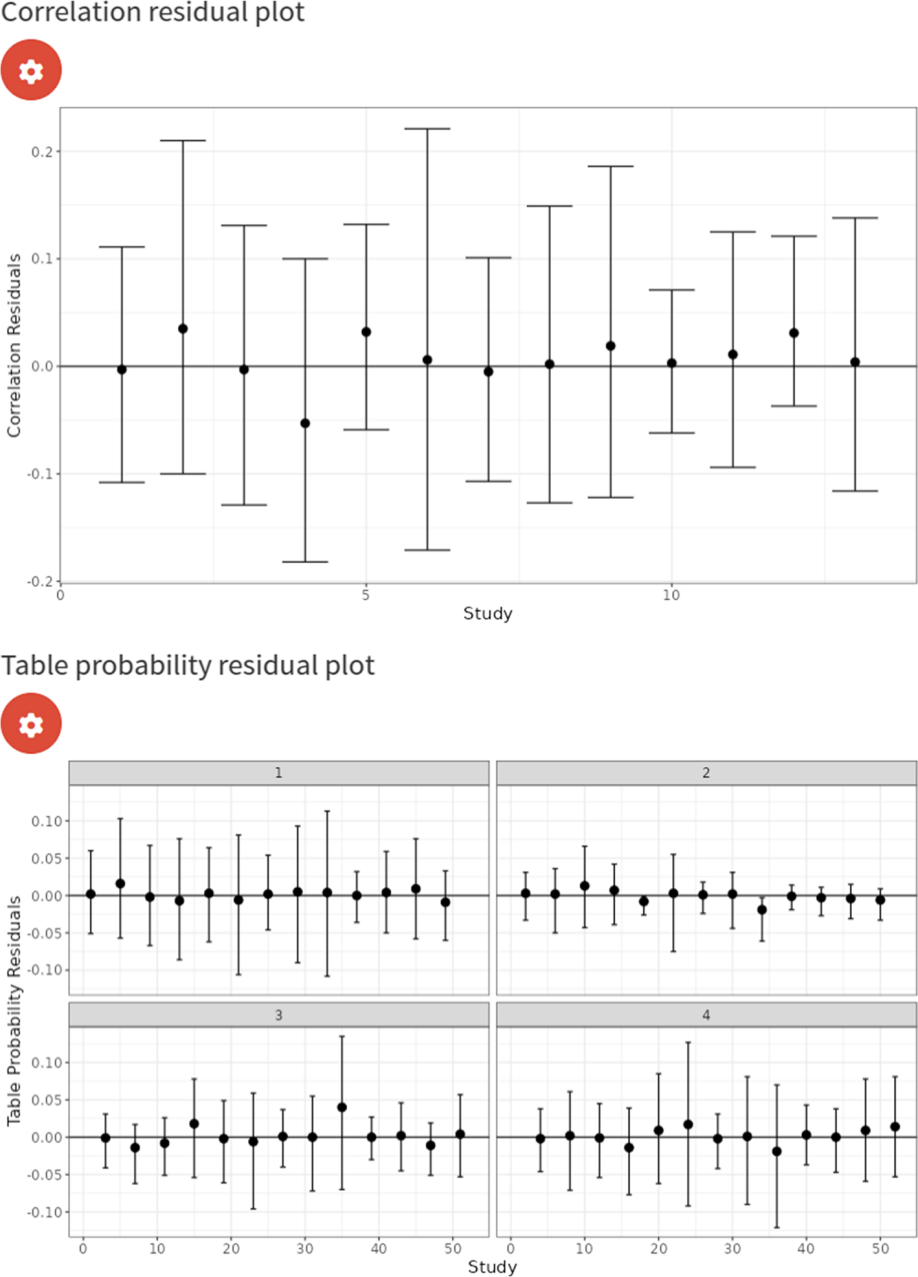
Correlation residual plot for LCM analysis, assuming conditional dependence between the IQCODE and reference tests, fixed effects for the reference tests, and random effects for the IQCODE. The red dots correspond to the study-specific estimates for the IQCODE

As previously mentioned, despite us obtaining a good correlation residual plot (see Fig. 11), the conditional independence assumption is typically not considered to be a reasonable assumption to make in clinical practice. Therefore, we attempted to fit a model without assuming conditional independence between the IQCODE and reference tests. Similarly to the conditional independence model, there was not enough information to identify all model parameters under the conditional dependence assumption if random effects were assumed for all tests (see supplementary material Fig. 5); therefore, we made the stronger assumption specifying fixed effects for the reference tests to identify the model. For the IQCODE, we obtained sensitivity and specificity estimates of 0.89 (95% CrI = (0.82, 0.95)) and 0.71 (95% CrI = (0.58, 0.84)). Both of these estimates are lower than the model assuming conditional independence (see “Analysis assuming conditional dependence” section), and suggest that the analysis assuming a perfect gold standard slightly overestimated the sensitivity by around 2% and underestimated the specificity by around 5%. An sROC plot of the results is shown in Fig. 12. Furthermore, although the model with conditional independence provided a satisfactory fit (see Fig. 11), the conditional dependence model clearly provides a better fit (see Fig. 13) - since it moves the summary estimates of the residual correlations and table frequency probability residuals closer to 0, and the median deviance has decreased from 54.8 to 43.8 (mean from 54.5 to 44.8).

## Discussion

In this paper, we presented MetaBayesDTA, an extensively expanded web-based R Shiny [17] application based on MetaDTA [9]. The application enables users to conduct Bayesian meta-analysis of diagnostic test accuracy studies, both assuming a perfect reference test or modelling an imperfect reference test, without users having to install any software or have any knowledge of R [16] or Stan [14] programming.

The application uses the bivariate model [1] to conduct analysis assuming a perfect reference test, and users can also conduct univariate meta-regression and subgroup analysis. It uses LCMs [4, 5] to conduct analyses without assuming a perfect gold standard, allowing the user to run models assuming conditional independence or dependence, options for whether to model the reference and index test sensitivities and specificities as fixed or random effects, and can model multiple reference tests using a meta-regression covariate for the type of reference test. The application allows users to input their own prior distributions, which is particularly useful for the LCM models since information about the accuracy of the reference test(s) is often known. Similarly to MetaDTA [9], the tables and figures can be downloaded, and the graphs are highly customizable. Furthermore, risk of bias and quality assessment results from the QUADAS-2 [22] tool can be incorporated into the sROC plot; integrating risk of bias into the main analysis decreases the tendency to think of risk of bias as an afterthought. Sensitivity analysis allowing users to remove selected studies can also be carried out easily for all models.

As we discussed in the “why is this application needed?” section (see Table 1), our app offers improvements over both BayesDTA [12] and MetaDTA [9–11]. Namely, for the bivariate model, unlike both BayesDTA and MetaDTA, our app allows subgroup analysis and univariate meta-regression (either categorical or continuous covariate) to be carried out, which also allows users to easily conduct comparative test accuracy meta-analysis to compare two or more tests to one another. Furthermore, unlike BayesDTA, for the LCM, our app can assess model fit using the correlation residual plot [30], and it can model multiple reference tests, using a categorical covariate for the type of reference test. This is important since studies included in meta-analysis of test accuracy often use different reference tests, and the accuracy can vary greatly between them. Even though it is more complicated than MetaDTA, as it can run 5 different models rather than one and the graphs have more customization options, it has a cleaner layout and many of the menus are hidden unless the user clicks on them to display more options, thanks to the shinydashboard [19] and shinyWidgets [20] R packages. In general, there are some benefits of using Bayesian methods for meta-analysis of test accuracy as opposed to frequentist. For instance, being able to include informative prior information is particularly useful for the imperfect gold standard model, where parameter identifiability if often an issue. Furthermore, Bayesian methods generally outperform frequentist methods when there are few studies in a meta-analysis (which is often the case) - as frequentist methods are more likely to underestimate the between-study heterogeneity [36].

Our web application has some limitations which give way to future developments. For example relating to meta-analysis of test accuracy without assuming a perfect gold standard (LCM), whilst users can model the data without assuming conditional independence between tests, it does not offer functionality to impose restrictions on the correlation structure. Therefore, a potential improvement would be to allow users to impose these restrictions, such as assuming the same correlation in the diseased and non-diseased groups, and/or forcing the correlations between the tests to be positive. Another limitation of the LCM is that it can only model different reference tests using categorical meta-regression and therefore assumes that all of the reference tests have the same between-study variances. Although this is often an advantage compared to conducting a subgroup analysis for each reference test, sometimes it might make sense to run a more complex model which assumes separate between-study variances for some reference tests and assumes fixed effects for reference tests only observed in a few (e.g., 5) studies, therefore adding this functionality is a potential update.

For the bivariate model, a potential update for both subgroup analysis and categorical meta-regression would be allow users to specify different priors for each of the groups. Furthermore, for meta-regression, although our application allows users to see the pairwise differences and ratio’s between the different categories of a categorical covariate (making it possible to use for comparative test accuracy of multiple tests), it only shows these for the meta-regression which assumes the variances are the same between all tests. However, in some instances it might make sense for the variances for some (or all - which would be equivalent to conducting a subgroup analysis) of the tests to be different, so a future update to improve the application would be to also display the pairwise differences and ratios for the subgroup analysis, and allowing users to assume independent variances for some tests but shared variances across other tests.

Another limitation is that our application only allows subgroup analysis and meta-regression (besides for modelling different reference tests) to be conducted using the bivariate model, which assumes a perfect gold standard. A potential improvement would be to allow users to run subgroup analyses and meta-regression for the LCM. Furthermore, the application requires users to have some knowledge about checking Bayesian model diagnostics to check that the models have been fitted OK - although the application does contain some information (in the “model diagnostics” tabs) which explains how to interpret some of the model diagnostics, and also directs users to online resources which explain how to interpret the model diagnostics so users do not have to find this information themselves.

It is important to note that this app is a beta version, so it is expected that there may be some bugs. Therefore, we welcome any user feedback - this can be done by completing the user feedback questionnaire (a link is provided in a pop-up box which appears when accessing MetaBayesDTA), or by emailing the first author of this paper. Responses to this feedback questionnaire will inform future updates of the application and will ensure that the user-friendliness of MetaBayesDTA increases over time and becomes a widely used diagnostic test accuracy meta-analysis web application, as MetaDTA [9] has become. A number of features included in MetaBayesDTA were included as a result of user and stake holder feedback - including the imperfect gold standard models, the meta-regression and subgroup analysis, the “hidden” menus and options to make the interface look cleaner and less intimidating, and the Bayesian capabilities of the application.

In general, one could argue that easy-to-use apps could lead to the over-application of complex methods even when they are not appropriate. This is because web applications - such as the one presented in this paper - will allow less experienced researchers to be able to conduct complex analyses which would otherwise be inaccessible to them, lowering the amount of knowledge needed to perform the analysis, and therefore increasing the chance of invalid results being published. Therefore, we recommend that there is a statistician (with knowledge of how to check Bayesian model diagnostics) in the review team. Furthermore, we have implemented a number of features in our application to minimise the risk of misleading research outputs being produced. These include: the informative pop-up boxes which appear which give information about setting up appropriate prior distributions and remind users to check the sampler diagnostics every time they run a new model, guidance in the “sampler diagnostics” tab so that users can interpret the sampler diagnostics, and implementing appropriate restrictions (e.g., whenever random-effects are used, the 95% prediction regions will always be displayed on the sROC plots - we do not allow only 95% credible regions to be displayed as this will not portray information about the between-study heterogeneity and can be misleading).

One could also argue that the widespread usability of apps could stimulate the uptake of more appropriate methods, which means that better methods will become standard practice more quickly. This could have important impacts for clinical practice; for instance, the fact that our app allows one to easily conduct a meta-analysis of test accuracy without assuming a gold standard without assuming the same reference test is used across all studies opens up many new datasets to synthesis, since many studies are conducted using different imperfect reference tests.

## Conclusions

In this paper, we presented MetaBayesDTA [13], a user-friendly, interactive web application which allows users to conduct Bayesian meta-analysis of test accuracy, with or without a gold standard. The application uses methods which were previously only available by using statistical programming languages, such as R [16].

This application could have a wide-ranging impact across academia, guideline writers, policy makers, and industry. For example, when there is not a perfect reference test available, the estimates of test accuracy can change quite notably when relaxing the perfect reference test assumption, leading to potentially different conclusions being drawn about the accuracy of a test which could ultimately lead to changes in which tests are used in clinical practice. Furthermore, the ability of the app to easily conduct comparative test accuracy meta-analysis means that clinicians will more easily be able to tell which tests are better.

## Availability and requirements

**Project name:** MetaBayesDTA

**Project home page:** https://crsu.shinyapps.io/MetaBayesDTA/

**Operating system(s):** Platform independent

**Programming language:** R, Stan

**Other requirements:** Web browser (R Shiny officially supports Google Chrome, Mozilla Firefox, Safari, or Internet Explorer)

**License:** Not applicable.

**Any restrictions to use by non-academics:** None

## Supplementary Information

**Additional file 1.**

## Data Availability

The web application (and the dataset used for analysis) is available at: https://crsu.shinyapps.io/MetaBayesDTA/. The data, R and Stan code for the web application is available at: https://github.com/CRSU-Apps/MetaBayesDTA.

## Abbreviations

HSROC: hierarchical summary receiver operating characteristic
LCM: latent class model
IQCODE: Informant Questionnaire on Cognitive Decline in the Elderly
QUADAS-2: QUality Assessment of Diagnostic Accuracy Studies, version 2
CI: Confidence Interval
sROC: summary Receiver Operating Characteristic
SD: Standard Deviation
LKJ: Lewandowski-Kurowicka-Joe
CrI: Credible Interval
DSM-III-R: Diagnostic and Statistical Manual of Mental Disorders version III, revised
DSM-IV: Diagnostic and Statistical Manual of Mental Disorders version IV
NINCDS-ADRDA: National Institute of Neurological and Communicative Diseases and Stroke/Alzheimer’s Disease and Related Disorders Association
ICD-10: International Classification of Diseases, version 10
