# Supplementary material for: MetaBayesDTA: codeless Bayesian meta-analysis of test accuracy, with or without a gold standard
Source: BMC Med Res Methodol. 2023 May 25;23:127. doi: 10.1186/s12874-023-01910-y (PMC10210277; doi:10.1186/s12874-023-01910-y)
Supplement: Supplementary file 1 — Additional file 1. [file 12874_2023_1910_MOESM1_ESM.zip › Supplementary/Supp_material_figure_legends.docx]

**Supplementary material figure legends**

Supplementary material figure legend 1:

sROC plot for the meta-analysis (bivariate) model showing risk of bias and quality assessment information

Supplementary material figure legend 2:

Posterior density plots for model assuming random effects for index test (IQCODE) and fixed-effects for reference tests, and conditional independence (CI) between tests.

Supplementary material figure legend 3:

Trace plots for model assuming random effects for index test (IQCODE) and fixed-effects for reference tests, and conditional independence (CI) between tests.

Supplementary material figure legend 4:

Posterior density plots for model assuming random effects for both the index test (IQCODE) and reference tests, and conditional independence (CI) between tests.

Supplementary material figure legend 5:

Posterior density plots for model assuming random effects for both the index test (IQCODE),and reference tests, and conditional dependence (CD) between tests.
