# Supplementary figures and images for: MetaBayesDTA: codeless Bayesian meta-analysis of test accuracy, with or without a gold standard
Source: BMC Med Res Methodol. 2023 May 25;23:127. doi: 10.1186/s12874-023-01910-y (PMC10210277; doi:10.1186/s12874-023-01910-y)

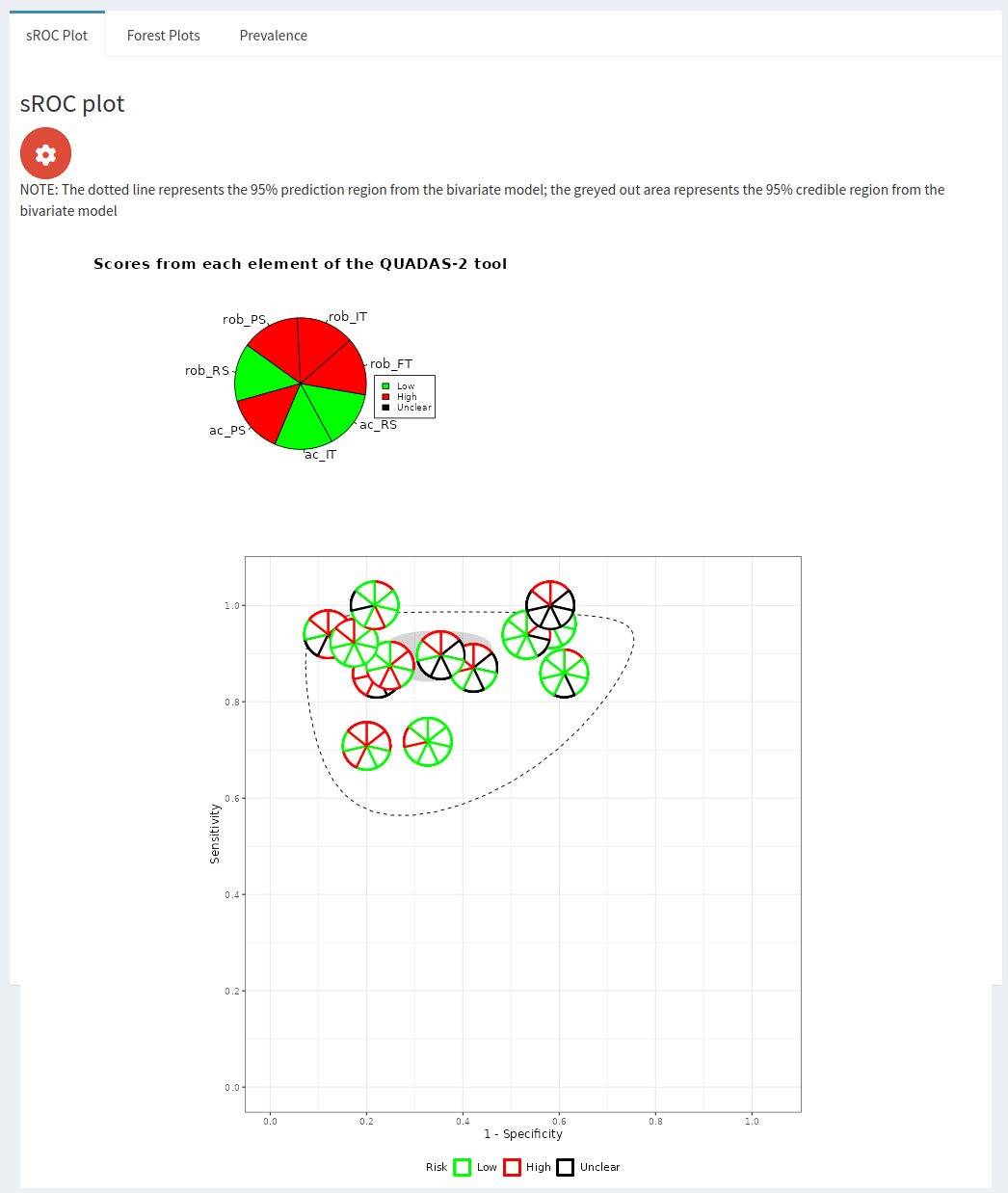

Supplement: Supplementary file 1 — Additional file 1. [file 12874_2023_1910_MOESM1_ESM.zip › Supplementary/Supp_mat_Fig_1_perfect_gs_meta_analysis_sroc_plot_rob_and_ac_pie_charts.png]

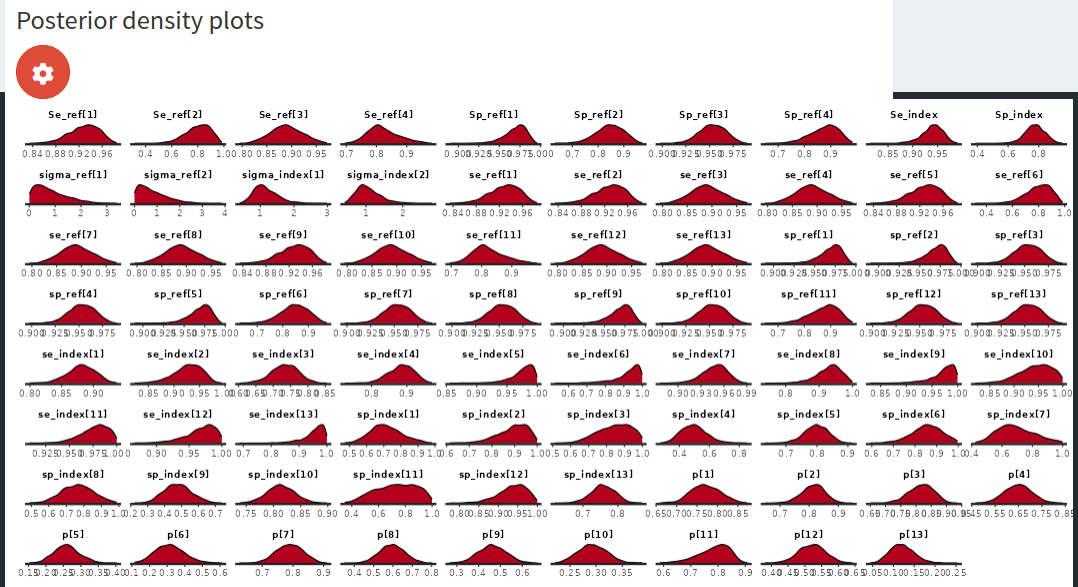

Supplement: Supplementary file 1 — Additional file 1. [file 12874_2023_1910_MOESM1_ESM.zip › Supplementary/Supp_mat_Fig_2_imperfect_gs_CI_fixed_ref_posterior_density_plots.png]

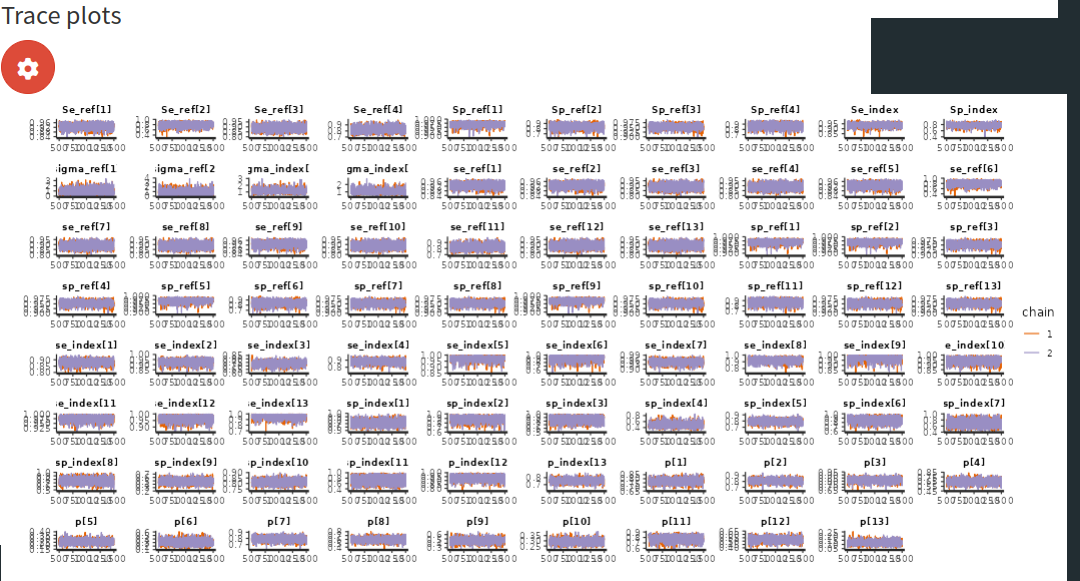

Supplement: Supplementary file 1 — Additional file 1. [file 12874_2023_1910_MOESM1_ESM.zip › Supplementary/Supp_mat_Fig_3_imperfect_gs_CI_fixed_ref_trace_plots.png]

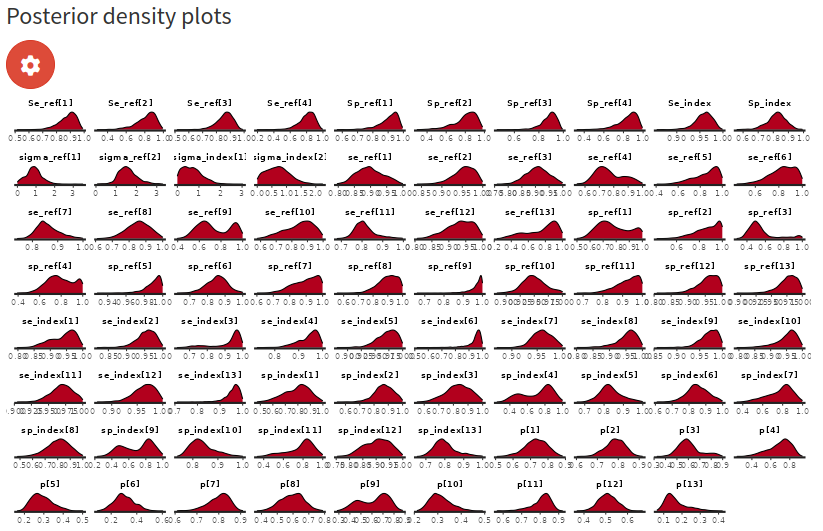

Supplement: Supplementary file 1 — Additional file 1. [file 12874_2023_1910_MOESM1_ESM.zip › Supplementary/Supp_mat_Fig_4_imperfect_gs_CI_posterior_density_plots.png]

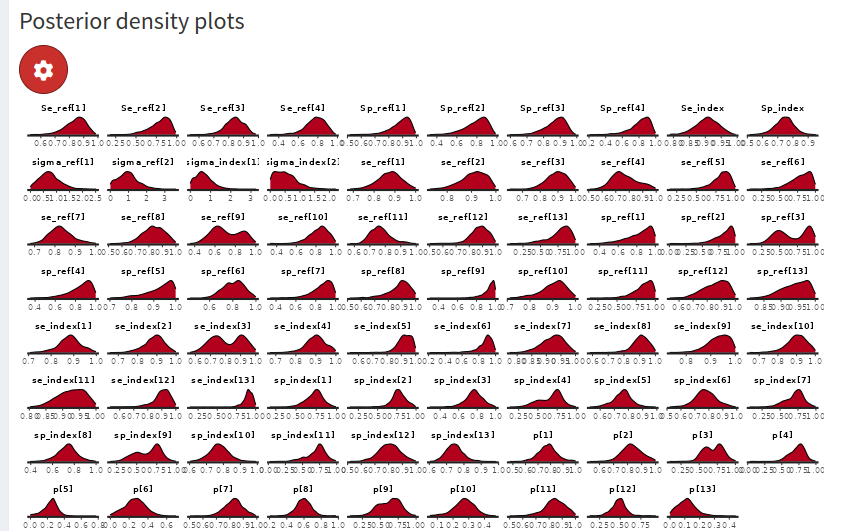

Supplement: Supplementary file 1 — Additional file 1. [file 12874_2023_1910_MOESM1_ESM.zip › Supplementary/Supp_mat_Fig_5_imperfect_gs_CD_posterior_density_plots.png]
